# Integrative genomic analysis of CREB defines a critical role for transcription factor networks in mediating the fed/fasted switch in liver

**DOI:** 10.1186/1471-2164-14-337

**Published:** 2013-05-17

**Authors:** Logan J Everett, John Le Lay, Sabina Lukovac, Diana Bernstein, David J Steger, Mitchell A Lazar, Klaus H Kaestner

**Affiliations:** 1 Institute for Diabetes, Obesity, and Metabolism, Perelman School of Medicine at the University of Pennsylvania, Philadelphia, PA, USA; 2 Department of Genetics, Perelman School of Medicine at the University of Pennsylvania, Philadelphia, PA, USA; 3 Division of Endocrinology, Diabetes, and Metabolism, Perelman School of Medicine at the University of Pennsylvania, Philadelphia, PA, USA

**Keywords:** CREB, Gene regulation, Hepatic fasting response, Integrative genomics, Transcription networks

## Abstract

**Background:**

Metabolic homeostasis in mammals critically depends on the regulation of fasting-induced genes by CREB in the liver. Previous genome-wide analysis has shown that only a small percentage of CREB target genes are induced in response to fasting-associated signaling pathways. The precise molecular mechanisms by which CREB specifically targets these genes in response to alternating hormonal cues remain to be elucidated*.*

**Results:**

We performed chromatin immunoprecipitation coupled to high-throughput sequencing of CREB in livers from both fasted and re-fed mice. In order to quantitatively compare the extent of CREB-DNA interactions genome-wide between these two physiological conditions we developed a novel, robust analysis method, termed the ‘single sample independence’ (SSI) test that greatly reduced the number of false-positive peaks. We found that CREB remains constitutively bound to its target genes in the liver regardless of the metabolic state. Integration of the CREB cistrome with expression microarrays of fasted and re-fed mouse livers and ChIP-seq data for additional transcription factors revealed that the gene expression switches between the two metabolic states are associated with co-localization of additional transcription factors at CREB sites.

**Conclusions:**

Our results support a model in which CREB is constitutively bound to thousands of target genes, and combinatorial interactions between DNA-binding factors are necessary to achieve the specific transcriptional response of the liver to fasting. Furthermore, our genome-wide analysis identifies thousands of novel CREB target genes in liver, and suggests a previously unknown role for CREB in regulating ER stress genes in response to nutrient influx.

## Background

The cAMP-response element binding protein (CREB) is a highly conserved effector of transcriptional changes in response to cAMP signaling in a wide range of physiological processes and cell types [1-4]. Increased cAMP levels cause activation of Protein Kinase A (PKA), which phosphorylates CREB on Serine 133 (S133), thereby promoting interactions with the co-activators and histone acetyltransferases CREB-binding protein (CREBBP), E1A binding protein p300 (EP300), and CREB-regulated transcription co-activator 2 (CRTC2) [1,5]. Although CREB binding and phosphorylation has been observed at thousands of genes, the majority of CREB target genes are not induced by cAMP *in vivo*[6]. *In vitro* studies have suggested that phosphorylation of S133 also promotes CREB binding to specific DNA sequences [7], providing a potential mechanism by which cAMP induces the expression of some CREB target genes, but not others. Supporting this notion, a small number of CREB target genes were shown to exhibit increased binding in the liver of fasted animals [8]. However, CREB binding dynamics have not been explored genome-wide, and the precise molecular mechanisms that direct this PKA-dependent activation signal to a subset of CREB-bound genes in a context-specific manner remain to be elucidated.

In the liver, CREB plays a central role in the maintenance of glucose homeostasis, and dysregulation of the CREB-dependent gluconeogenic gene program is a contributing factor in a number of metabolic diseases including type 2 diabetes [9-12]. A drop in blood glucose levels triggers a glucagon/epinephrine-dependent signaling cascade resulting in the cAMP-induced phosphorylation of CREB S133 in hepatocytes to induce the expression of key gluconeogenic genes [1,13]. Previous genome-wide studies in primary hepatocytes have shown that CREB binds to thousands of target gene promoters, while only a small subset of these genes are induced by cAMP [6,14]. However, these studies did not examine changes in CREB DNA binding and did not identify features separating cAMP-inducible and non-inducible CREB targets. Furthermore, these studies used chromatin immunoprecipitation coupled to genomic microarrays (ChIP-chip) technology, which was limited to the promoter regions of known genes and showed limited sensitivity even within these regions.

To address the question of whether CREB DNA binding dynamics play a role in fasting-induced gene expression, we performed chromatin immunoprecipitation coupled to high-throughput sequencing (ChIP-seq) and gene expression analysis by microarray on mouse livers in both fasted (high glucagon/insulin ratio) and re-fed conditions (low glucagon/insulin ratio). Controlled fasting and re-feeding has been shown to be a robust model of cAMP-dependent CREB activity in the mammalian liver [15,16], and ChIP-seq provides a major improvement in the sensitivity and resolution of CREB binding site detection compared to previous ChIP-chip studies. Our results demonstrate that global CREB DNA binding in the liver is independent of cAMP/PKA-signaling, and support a model in which CREB remains constitutively bound to DNA irrespective of its phosphorylation state. Strikingly, we also discovered CREB binding at thousands of novel target genes, including genes that are transcriptionally repressed in the fasted liver versus the re-fed liver. To further elucidate how CREB specifically regulates subsets of target genes, we performed an integrative computational analysis of the CREB cistrome and fasting-dependent transcriptome in mouse livers to identify genomic features correlated with CREB sites at fasting-responsive genes. In particular, we observed a strong enrichment for binding of additional transcription factors proximal to the CREB sites specifically at fasting-responsive genes, suggesting that the cooperation between transcriptional regulators at the level of individual regulatory elements — as opposed to entire promoters — is more widespread and critical to gene induction than previously demonstrated. Our work represents an integrative paradigm for studying the context-dependent role of a transcriptional regulator in a specific physiological process.

## Results

### Genome-wide CREB-DNA binding is independent of cAMP/PKA-signaling

Two models have been proposed as to how CREB contributes to gene activation in response to cAMP stimuli. Early studies found CRE “half-sites”, composed of only five of the eight nucleotides of the consensus CRE, were bound more tightly by CREB phosphorylated on S133 than by unphosphorylated CREB *in vitro*[7], suggesting that partial CRE motifs play a critical role in cAMP-dependent gene induction. *In vivo* studies have observed both dynamic [8] and constitutive CREB binding to DNA [17,18], but these studies only tested a small subset of known CREB target genes. Genome-wide studies of CREB in hepatocytes have not examined CREB DNA binding dynamics, but have shown that CREB binds a large proportion of gene promoters that are not induced by cAMP [6,14]. We therefore began our investigation by examining the genome-wide profiles of CREB binding to DNA in response to fasting-induced cAMP signaling to test comprehensively if site-specific dynamic CREB binding could be observed *in vivo*.

**Figure 1:**
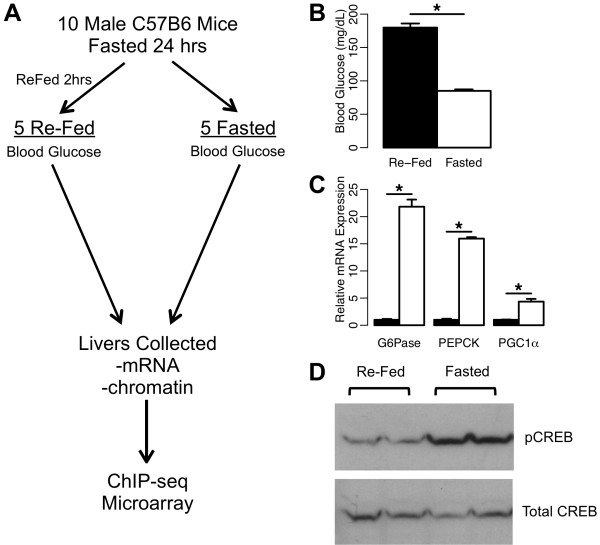
**Experimental design and validation. A**) Overview of experimental design. A cohort of 10 male C57BL/6J mice was fasted for 24 hours, then split into fasted and re-fed groups (n=5 each). The fasted mice were immediately sacrificed, while the re-fed group was given access to food for 2 hours before sacrificing. Blood glucose measurements were taken from all mice prior to sacrifice. Livers were collected shortly after sacrifice, and used for both mRNA and ChIP-seq experiments. **B**) Blood glucose was significantly higher in re-fed animals. Error bars indicate SEM, *p < 1E-4, one-tailed Student’s *t*-test. **C**) Known fasting-induced gluconeogenic genes are significantly induced in the fasted state (white bars) relative to the re-fed state (black bars) by RT-PCR. Error bars indicate SEM, *p < 0.005, one-tailed Student’s *t*-test). **D**) Western blot analysis of mouse livers shows that CREB is strongly phosphorylated on Ser133 after a 24 hr fast, but not after 2 hour re-feeding. Western blot was re-probed with an antibody for total CREB to validate equal loading.

In order to quantitatively test genome-wide binding of CREB in both the fasted (cAMP/PKA-active) and re-fed (cAMP/PKA-inactive) states, we performed a controlled feeding experiment (Figure 1A) in which 10 mice were fasted for 24 hours, and either immediately sacrificed (fasted group), or fed again for two hours before sacrificing (re-fed group). We observed a robust and consistent decrease in blood glucose for the fasted mice relative to the re-fed mice (Figure 1B), and RT-PCR measurements confirmed induction of well-characterized cAMP-responsive genes *G6pc*[19], *Pck1*[20,21], and *Ppargc1a*[13] in the livers of fasted mice (Figure 1C). Western blot analysis showed that CREB phosphorylation at S133, indicating activation of the cAMP/PKA pathway, was also specific to the fasted state as expected and not to the re-fed state (Figure 1D), in contrast to what has been reported previously after acute insulin injections intended to mimic the re-fed state [5]. Liver chromatin from individual mice was subsequently used to perform ChIP-seq with an antibody recognizing CREB, regardless of phosphorylation status.

The large number of biological replicates included in our ChIP-seq study allowed us to apply a novel peak-calling and filtering strategy that removes non-reproducible peaks, while maintaining detection sensitivity across a wide range of enrichment strengths. Under our strategy, termed the “Single-Sample Independence (SSI) test”, sequence reads from all five fasted or re-fed replicates were first pooled together and used in peak-calling to identify all candidate CREB binding events. This was followed by a filtering step in which each peak was retained only if a similar peak was called after excluding any individual replicate from the pool of five replicates (Additional file 1: Figure S1A). This filtering strategy removed greater than 50% of the initial peak calls, and resulted in a much stricter set of target sites than would be obtained by simply applying a more conservative false discovery rate cutoff to the initial candidate peak calls, while also taking advantage of the biological replicate structure of the data set (Additional file 1: Table S1). The peak calls eliminated by our SSI strategy typically occurred at sites inconsistent with known CREB biology in terms of the occurrence of known motifs and bias toward promoter regions, showed less overlap between the fasted and re-fed states, less overlap with ENCODE DNaseI-hypersensitive (DHS) regions [22,23] (Additional file 1: Figure S1B, HOMER columns), and tended to fall in a weaker range of peak heights below 0.5 reads per million (RPM) (Additional file 1: Figure S1C).

The SSI strategy can be applied using any peak-calling algorithm; we tested the SSI filter using the previously published peak-calling tools HOMER [24], GLITR [25], and MACS [26]. While the results were comparable using any of these peak-calling algorithms, the HOMER+SSI combination resulted in the smallest number of algorithm-specific peaks, and the set of sites with the highest occurrence of known CREB motifs (Additional file 1: Figure S1B,D). We also assessed the number of peaks filtered out after excluding each individual replicate and found a similar percentage of sites are dependent on each replicate, confirming that each replicate is of comparable quality (Additional file 1: Figure S1E). Therefore, the peaks called by HOMER+SSI using all replicates were used for further analysis.

We further validated our SSI-filtered peak calls by performing ChIP-qPCR on randomly selected peaks across the range of peak heights. Importantly, these validation experiments showed that peaks with an average peak height below 0.35 RPM (the bottom quartile of SSI-filtered peaks) were not reproducible, while peaks above this cutoff had a high rate of reproducibility (Additional file 1: Figure S2). Furthermore, 96% of the peaks above this cutoff overlapped ENCODE DHS regions in mouse liver [22,23], suggesting that the majority of these sites represent real binding events. Overall, our extensive peak-filtering strategy identified 6,835 and 5,357 high-confidence CREB binding sites in the fasted and re-fed states, respectively.

**Figure 2:**
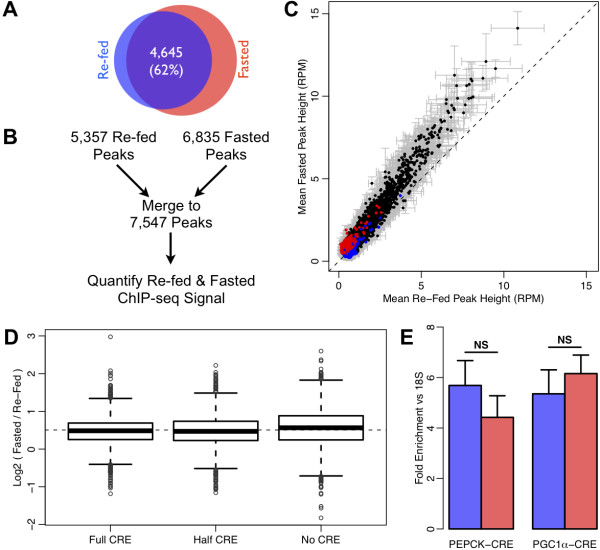
**Differential CREB binding analysis. A**) Venn diagram of high-confidence CREB site calls in the re-fed (blue) and fasted (red) mouse liver suggests thousands of condition-specific binding events. **B**) Flowchart of quantitative analysis. **C**) Scatter-plot of average peak height (RPM = reads per million) across all fasted and re-fed replicates, for 7,547 merged peaks. Black dots correspond to peaks called in both conditions, red dots correspond to peaks called only in the fasted group, and blue dots correspond to peaks called only in the re-fed group. Gray error bars indicate SEM. **D**) Boxplot of log2 fasted/re-fed ratio of peak height for subsets of CREB binding sites based on occurrence of canonical full or half CRE sequence within 50 bp of peak center. **E**) ChIP-qPCR of CREB binding in the re-fed (blue) and fasted (red) groups on known fasting-induced CREB target genes. Neither change is significant at p-value of 0.05 threshold (one-tailed Student’s *t*-test).

Our high-confidence peak calls from the fasted and re-fed replicates exhibited 62% overlap (Figure 2A), at first glance suggesting that thousands of CREB binding sites may depend on CREB phosphorylation level, as proposed originally by Nichols and colleagues [7]. However, the absence of a peak call in one condition, though frequently employed in the literature [27-29], is not a sufficient criterion for demonstrating differential binding, because peak-calling algorithms provide no estimate of the false negative rate. In other words, these apparently “differentially bound” regions may actually indicate cases in which the peak call was simply missed in one of the two conditions. To address this issue directly, we merged together all fasted and re-fed peak calls to obtain a total of 7,547 high-confidence CREB binding sites, and quantified the extent of ChIP-seq signal at each site, in each replicate, under each condition (Figure 2B). As revealed by the scatterplot of average fasted versus average re-fed ChIP-seq peak height (Figure 2C), the strength of CREB binding at any site was highly comparable between the two conditions, with only a weak global induction (median = 1.3-fold) of CREB binding in the fasted state. Sites that were originally specified as condition-specific occurred predominantly in the lower range of peak heights, where false negative calls by peak-calling algorithms are most likely, and the peak strength in the other condition tended to be comparable despite the lack of a complementary site call. Furthermore, we observed a less than a 1.1-fold difference in the median fasting/re-fed CREB binding ratio when comparing subsets of CREB sites separated by CRE motif occurrence (Figure 2D), further contradicting the model proposed by Nichols and colleagues [7].

Targeted ChIP-qPCR of CREB binding sites around the known cAMP-inducible genes *Pck1* and *Ppargc1a* in both metabolic states confirmed the constitutive binding pattern observed in our ChIP-seq experiment (Figure 2E). We also analyzed individual peaks for differential binding across conditions using the EdgeR tool [30], but were only able to identify 12 regions (0.2% of total) with significant differential CREB enrichment at a 10% FDR threshold (Additional file 1: Table S2). Of these 12 differential peaks, 3 were greater than 10 kb away from any known genes, and another 3 were within 500 bp of a separate peak that did not pass the EdgeR 10% FDR threshold for differential enrichment. Therefore, we conclude that differential binding of CREB in response to cAMP-dependent phosphorylation does not play a critical genome-wide role in fasting-induced gene expression.

Our quantitative analysis demonstrates that total CREB binding to DNA in liver is, at most, only weakly induced by fasting-dependent signaling, with minimal sequence or site specificity. Given that we observed substantially larger differences in target gene expression in the same experiment (Figure 1C), it is highly unlikely that dynamic binding at specific sites is the primary regulatory mechanism for the specific induction of cAMP-responsive genes by CREB *in vivo*.

### CREB ChIP-seq in liver reveals novel target genes and binding elements

**Figure 3:**
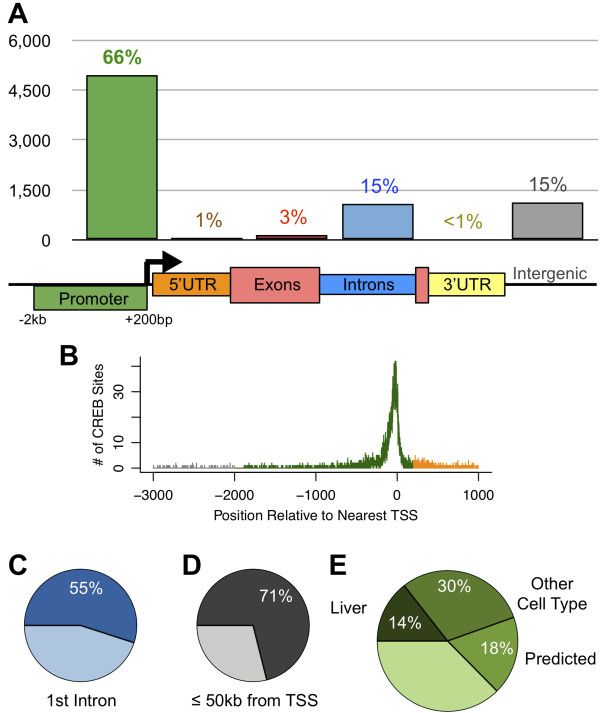
**Hepatic CREB binding relative to gene structure. A**) The proportion of 7,547 high-confidence CREB binding sites with peak center in promoter regions (green, defined as -2 kb to +200 bp around TSS), 5’ UTR (orange), exons (red), introns (blue), and 3’ UTR (yellow). The remaining sites are considered intergenic (gray). **B**) Frequency of CREB binding site positions relative to known TSS. Colors correspond to feature definitions used in (**A**). **C**) Proportion of intronic CREB sites occurring in the first intron of a known transcript. **D**) Proportion of intergenic CREB sites occurring within 50 kb of a known TSS. **E**) We identified 7,095 genes bound by CREB at distal, proximal, or internal sites based on our high-confidence ChIP-seq peaks. Pie chart shows the proportion of target genes previously identified in liver cells [6], other mammalian cell types [6,32], or predicted by bioinformatics analysis [6]. The remaining 2,641 CREB-bound genes were not identified or predicted in the previous genome-wide studies.

Having established that CREB binding to its target genes is largely independent of its activation state in the liver, we used the 7,547 CREB binding sites identified across both physiological conditions to further investigate the hepatic CREB cistrome. Reassuringly, our high-confidence peak calls include binding sites corresponding to many known target genes, including *Pck1*, *Ppargc1a*, and *Crem* (Additional file 1: Figure S3). We compared the hepatic CREB cistrome to all known gene loci and found that CREB binding is highly biased towards known promoter regions (66% of sites), with particular enrichment of sites 500 bp upstream to 200 bp downstream of transcription start sites (TSS) (Figure 3A,B). CREB binding events in the remainder of the 5’ UTR (greater than 200 bp downstream of TSS), coding exons, or 3’ UTRs were extremely rare. A substantial portion of the remaining sites (15% of total) fell into intronic regions, with more than half of these events occurring in the first intron of a known transcript (Figure 3A,C). The remaining CREB sites (15%) were intergenic, with the majority of these sites within 50 kb of a known TSS (Figure 3A,D). A previous analysis of the CREB cistrome in rat neurons using serial analysis of chromatin occupancy (SACO) had suggested a much higher percentage of intergenic sites [31]. This difference can be explained in part by the limited gene annotations available at the time of the prior study, as well as differences in the cell type used and in the higher accuracy of ChIP-seq versus SACO. A more recent ChIP-seq study of CREB binding in murine male germ-line cells found a similar number of bound sites and similar rates of promoter and intron overlap as our liver cistrome [32]. Overall, our analysis suggests that in the liver most CREB sites are linked to the regulation of known genes via upstream or internal promoters.

We considered any gene with a CREB peak inside the gene body or up to 10 kb upstream of the TSS to be a CREB target gene in liver, resulting in a total of 7,095 target genes that we compared to previous genome-wide studies (Figure 3E). Strikingly, only 14% of these target genes were previously observed as bound by CREB in a ChIP-chip study of human hepatocytes [6], even after mapping gene regions between these studies to remove bias from updated or species-specific gene annotations. 30% of the target genes in our analysis were observed in other mammalian cell types but not hepatocytes [6,32], and 18% were predicted only by bioinformatic sequence analysis but never demonstrated by *in vivo* genome-wide experiments [6]. A substantial portion of target genes (37.4%) had never been observed or predicted as CREB-bound in previous genome-wide studies.

**Figure 4:**
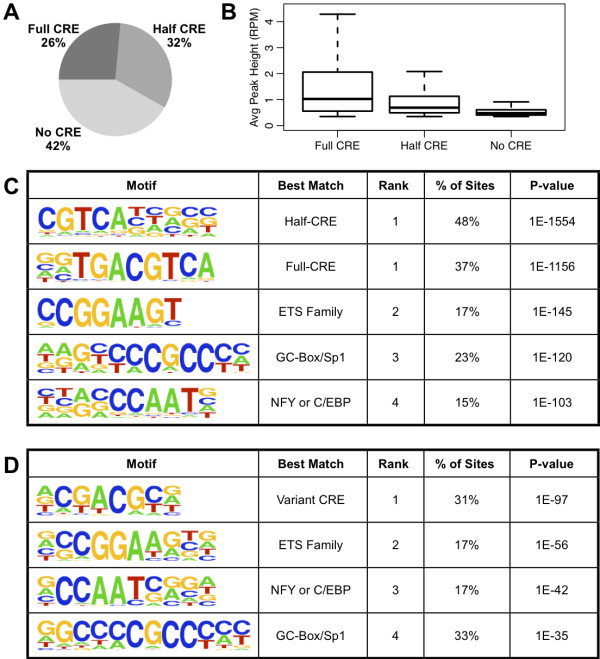
**Motif analysis of CREB binding sites. A)** Percentage of binding sites containing a Full CRE (‘TGACGTCA’ with at most one mismatch), Half CRE only (‘TGACG’ exact match), or neither type of CRE within +/- 50 bp of peak center. **B)** Distribution of peak heights within each category of site identified by sequence content in (A). **C)** Top five *de novo* motifs returned by HOMER for the 7,547 high-confidence CREB binding sites in the liver. Sites with the same rank were clustered together as similar motifs by HOMER. Percentage of binding sites and P-value were calculated using the optimal motif score threshold as determined by HOMER. **D)** Top *de novo* motifs returned by HOMER for the 3,155 high-confidence CREB binding sites lacking a canonical CRE sequence in (A).

To assess the motif content of the CREB cistrome, we first searched for instances of the canonical CRE motif within +/−50 bp of peak centers. We considered matches to the 8mer ‘TGACGTCA’ with up to 1 mismatched nucleotide to be “Full CRE” sequences, and exact matches to the 5mer ‘TGACG’ to be “Half CRE” sequences. These sequences were found under the majority of our peaks, although surprisingly more than a third of all high-confidence CREB sites lacked an apparent CRE motif (Figure 4A). The strength of CREB binding generally depends on this primary motif, as sites containing a Full CRE have a higher distribution of peak heights and sites lacking any CRE have a lower distribution of peak heights (Figure 4B).

*De novo* motif analysis of the CREB cistrome confirmed the enrichment of Full and Half CRE sequences as the top motifs, along with several other known promoter features such as GC-Box and ‘CCAAT’ motifs (Figure 4C). To further investigate the sites lacking canonical binding sequences, we repeated the *de novo* motif analysis on only those sites without a CRE sequence match. We found a more degenerate CRE sequence as the top motif among these sites, but otherwise revealed the same motifs as the whole cistrome analysis, although the GC-Box motif was more enriched among these sites (Figure 4D). Positional enrichment analysis showed that the canonical CRE motifs are highly biased towards peak centers, but show no enrichment in the regions immediately flanking the peaks, as expected for a primary binding motif (Additional file 1: Figure S4A,B). Interestingly, the degenerate CRE also shows specificity for peak centers, and is more enriched among the sites lacking a canonical CRE, suggesting that these degenerate consensus sequences are still directly recognized by the CREB protein (Additional file 1: Figure S4C). The additional *de novo* motifs corresponding to other DNA-binding factors also show positional bias around CREB sites, although they tend to be more widely distributed compared to CRE motifs (Additional file 1: Figure S4D-F). Overall, these results suggest that CREB binding to DNA is more promiscuous than previously appreciated, and CREB may cooperatively or indirectly bind DNA with additional factors at a subset of target sites.

### CREB binding alone is insufficient to confer fasting-responsiveness to target genes

**Figure 5:**
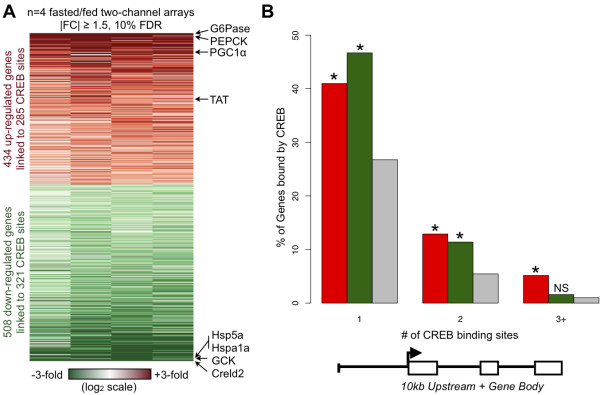
**Enrichment of CREB binding around fasting-responsive genes. A**) Heatmap of genes called as differentially expressed in fasted versus re-fed liver microarray experiment. Known fasting-inducible CREB targets are shown on top right. Selected feeding-inducible and ER stress genes are shown at bottom right. Number of genes and associated CREB sites are shown on left. **B**) For each gene category (fasting-induced in red, fasting-repressed in green, and all genes on the microarray in gray), we computed the percentage of genes containing 1, 2, or 3+ high-confidence CREB binding sites anywhere in the 10 kb TSS upstream region or the gene body (including introns). ~60% of regulated genes (both fasting-induced and fasting-repressed) have at least one CREB binding site by this criteria, while 33% of all genes on the array can be associated with at least one CREB binding site. *p-value < 1E-5 by Fisher’s Exact Test versus gray bars. NS indicates p-value > 0.05. A full listing of differentially expressed genes and associated CREB binding is provided in Additional file 2.

Given that our CREB ChIP-seq data indicated very few significant changes in target occupancy in response to the fasting-induced cAMP/PKA signal, we next used expression microarrays to identify which CREB targets are induced during fasting. We directly compared gene expression patterns between mouse livers in the fasted and re-fed conditions, and observed significant changes in 942 genes, including the aforementioned cAMP-inducible targets *Pck1*, *Ppargc1a*, and *G6pc* (Figure 5A, Additional file 2). We also observed reduced expression of many ER stress genes, such as *Hspa5* (*Grp78/Bip*), *Hspa1a*, and *Creld2*, in the fasted state relative to the re-fed state, in agreement with previous findings that nutrient influx during re-feeding induces mild ER stress [33,34].

We next compared our expression microarray results to the high-confidence CREB binding sites determined by our ChIP-seq analysis. We found that, in general, both genes that are induced or repressed in the fasted state are enriched for nearby CREB binding (Figure 5B; Additional file 1: Figure S5). The enrichment of CREB binding around fasting-repressed sites is particularly surprising, given that CREB has been reported to function solely as an activator of gene expression [1]. In total, 285 (4%) CREB binding sites were associated with fasting-induced genes, and therefore considered to be the most likely cAMP-responsive sites in liver. Another 321 (4%) sites were associated with fasting-repressed genes, and 6,018 (80%) sites were associated with genes not significantly altered in our microarray experiment. Thus, it appears that only a small subset of CREB binding sites are involved in conveying the cAMP signal to control gene expression in the liver, despite the observation that the majority of CREB sites are in known promoter regions (Figure 3A,B). Given that CREB-DNA interactions do not change significantly at the majority (>99%) of sites in response to cAMP signaling (Figure 2, Additional file 1: Table S2), and that the co-activators CREBBP, EP300, and CRTC2 have no inherent sequence specificity, this result strongly supports the conclusion that other genomic features, in addition to the presence of CREB binding, are required for cAMP-responsive changes in gene expression.

**Figure 6:**
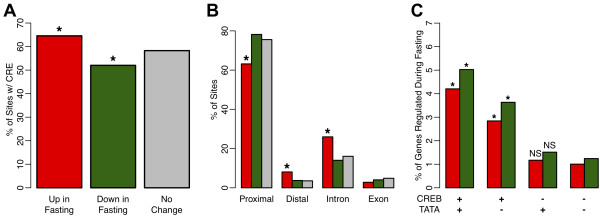
**Association of CREB binding sites with fasting-responsive TATA-box containing promoters.** CREB sites were separated into 3 groups: 285 sites associated with genes up-regulated in fasted state (red), 321 sites associated with genes down-regulated in fasted state (green), and 6,018 sites associated with genes that did not change (gray). **A**) Rates of CRE motif sequences (either full CRE or half CRE) identified for each group of CREB sites. *p-value < 0.05 by Fisher’s exact test versus the ‘No Change’ group. **B**) Rates of CREB sites, separated by association with fasting-dependent regulation, occurring in proximal promoter (−2 kb to +200 bp around TSS), distal promoter (−10 kb to -2 kb upstream of TSS), intronic, and exonic regions. *p-value < 0.001 by Fisher’s exact test compared to corresponding gray bar. **C**) All genes on our microarray were separated into groups based on associated CREB binding and presence of a TATA-box in the promoter. For each group, the percentage of genes induced (red) and repressed (green) in response to fasting is shown. *p-value < 1E-11, by Fisher’s Exact Test against the CREB-/TATA- group. NS indicates p-value > 0.05.

To investigate the mechanism of specificity of the cAMP-response, we tested for the enrichment of various genomic features within the three subsets of CREB sites associated with fasting-induced, repressed, or non-responsive genes defined above. We found only minor differences in the occurrence of CRE motifs and intronic versus promoter positioning of sites (Figure 6A,B). Thus, differences in the sequence content and relative positioning of CREB binding sites do not explain the specificity of the observed expression response as had been proposed previously [6,35].

Previous results on a small set of known targets had suggested that only promoters containing a TATA-box are inducible by a CRE site [18]. We classified all gene promoters in our analysis as TATA-containing or TATA-less, and again found only a weak association with fasting-responsive CREB targets. Specifically, while CREB-bound TATA-containing genes are the most likely to be induced by fasting, CREB-bound TATA-less genes are also significantly enriched for fasting-responsive genes, and CREB-bound TATA-containing genes are equally enriched among fasting-repressed genes (Figure 6C), suggesting that the mechanism for specificity is more complex than previously proposed.

### Combinatorial transcription factor binding determines gene regulation in response to the fasting-feeding switch in the liver

Based on the results above, we hypothesized that additional sequence-specific transcription factors may contribute to the specificity of the fasting response at a subset of CREB sites. Previous work, focused on several well-studied promoters, had shown synergistic effects of CRE sequences with other transcription factor binding sites [8,20,36,37]. We therefore tested the enrichment of binding by other transcription factors involved in the hepatic fasting response around CREB binding sites. We used previously published ChIP-seq data for forkhead box protein A2 (FOXA2) [25,38] and peroxisome proliferator activated receptor alpha (PPARA) [39], and performed additional ChIP-seq experiments for the glucocorticoid receptor (NR3C1/GR) and CCAAT/enhancer-binding protein beta (CEBPB), as these factors have all been shown to play a major role in the response to fasting in mammalian liver [8,40-42].

**Figure 7:**
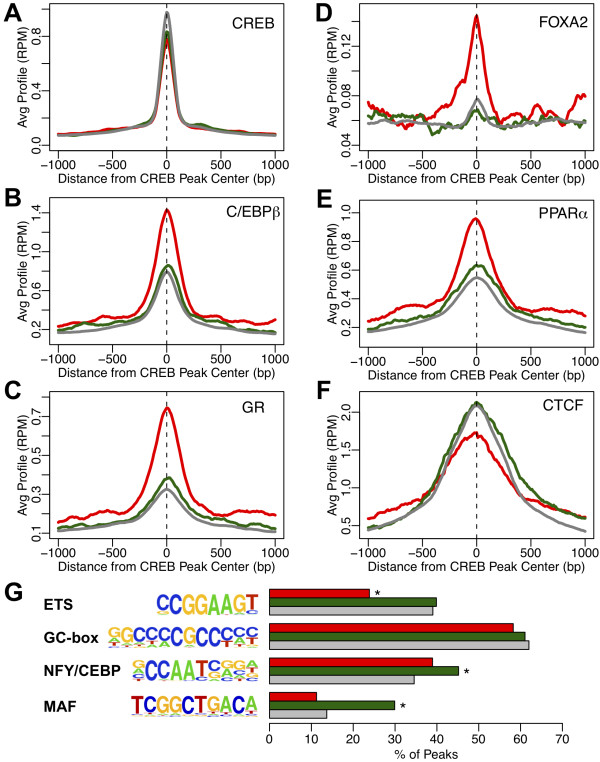
**Association of CREB binding sites with additional transcription factor occupancy at fasting-responsive genes.** CREB sites were separated into 3 groups: 285 sites associated with genes up-regulated in fasted state (red), 321 sites associated with genes down-regulated in fasted state (green), 6,018 sites associated with genes that did not change (gray). **A-F)** Average ChIP-seq profiles around each group of CREB binding sites are shown for CREB (**A**), CEBPB (**B**), NR3C1/GR (**C**), FOXA2 (**D**), PPARA (**E**), and CTCF (**F**). Corresponding distributions of peak heights are shown as box plots in Additional file 1: Figure S7 and tabulated in Additional file 3. **G**) Percentage of sites in each group containing the indicated motifs within +/− 250 bp of peak center. *p-value < 0.001 by Fisher’s exact test compared to gray bar.

The binding strength of CREB itself showed no association with the differential expression of the corresponding target gene in the hepatic feeding to fasting switch (Figure 7A, Additional file 1: Figures S6 and S7A, Additional file 3). The situation was strikingly different, however, for CEBPB, PPARA, NR3C1/GR and FOXA2, binding of all of which was much stronger near CREB binding sites associated with the genes induced in response to fasting compared to all others, with the greatest differential observed for FOXA2 (Figure 7B-E; Additional file 1: Figure S7B-E ). In contrast, binding of the Zinc-finger transcription factor CTCF in the mouse liver [22,23], while also generally enriched near CREB binding sites, did not correlate with the inducibility of CREB target genes after fasting (Figure 7F, Additional file 1: Figure S7F). Overall, our results suggest that the combinatorial action of a group of transcription factors is responsible for the induction of a small subset of CREB-bound promoters in the liver, and the simple presence of a CRE or even a proven CREB binding event is not sufficient to determine whether the associated gene is part of the fasting response.

We performed additional *de novo* motif analysis of CREB sites, directly comparing those sites associated with fasting-induced and -repressed genes to the non-responsive sites, and also computed the group-wise enrichment of the additional *de novo* motifs identified from the entire CREB cistrome (Figure 7G). We did not find any additional motifs specifically associated with fasting-induced sites, but we observed a significant enrichment for a motif at feeding-induced sites matching the half-site recognized by the Maf family of transcription factors [43]. We also observed that the ETS family motif is specifically depleted around CREB sites associated with fasting-induced genes, while the ‘CCAAT’ motif is slightly enriched at feeding-induced sites, although not to the same extent as the Maf family motif. Collectively, these results suggest that transcription factors from multiple families influence the direction of the CREB regulatory response to the feeding/fasting metabolic switch.

## Discussion

### Reproducible ChIP-seq analysis in a tightly controlled physiological system supports a model of constitutive CREB DNA binding

Here, we employed ChIP-seq in the context of a controlled fasting/re-feeding experiment — a robust model of CREB activation downstream of the cAMP/PKA pathway — to elucidate the role of CREB binding at specific sequence elements in the regulation of fasting-responsive genes in the liver. Our ChIP-seq data represent the first characterization of CREB binding events across the entire genome in the mammalian liver. Previous studies of CREB DNA binding in hepatocytes [6,14] were limited to known proximal promoter regions and did not explore changes in CREB occupancy. Our data reveal that CREB-DNA interactions in the liver, many of which occur at thousands of novel target genes missed by previous studies, are largely independent of CREB activation by the cAMP/PKA pathway *in vivo*, thus ruling out CREB DNA binding dynamics as a determinant of cAMP-responsive genes.

We performed our ChIP-seq experiment with a higher number of individual biological replicates than are typically used in genome-wide location analysis, and developed a novel peak-filtering strategy based on both computational analysis and independent experimental confirmation of reproducibility. In particular, our analysis demonstrates that a peak height cut-off is an important additional filter for ChIP-seq data, even when other stringent statistical criteria are applied. Notably, our empirically determined threshold was much lower than thresholds used in other ChIP-seq analyses [28], highlighting the variability of such attributes across different factors, antibodies, and sample material. Thus, each ChIP-seq study currently requires additional experimental validation to determine the optimal peak height threshold. Interestingly, our experimentally determined height cutoff (Additional file 1: Figure S2) corresponds closely to the crossover point between peak height distributions for those peaks passing or failing our SSI test (Additional file 1: Figure S1C), suggesting that our SSI test may provide a suitable model for determining the appropriate peak height cutoff without laborious ChIP-qPCR validation experiments.

We also demonstrated the importance of rigorous quantitative analysis methods when comparing ChIP-seq results across conditions. Using differences in peak calls alone, as is done in typical Venn diagrams comparing peaks called for a transcription factor or modified histone in two or more conditions [27-29], can dramatically over-state the differences between ChIP-seq profiles, and result in thousands of erroneous calls of differentially bound regions. In contrast, our quantitative analysis revealed that CREB ChIP-seq profiles in the re-fed and fasted states are tightly correlated genome-wide. Although our analysis cannot rule out a weak global induction of CREB-DNA binding after fasting, it is important to note that only a few individual sites (0.2%) showed a statistically significant difference in CREB occupancy between states (Additional file 1: Table S2). Of these sites, only one was near a fasting-induced gene (*Mt1*) on our microarray. Similarly, there was no significant difference in binding for our ChIP-qPCR experiments for cAMP/fasting-inducible CREB targets (Figure 2E), confirming that our ChIP-seq analysis was unlikely to be confounded by a lack of sensitivity or quantitative accuracy. Therefore, we conclude that differential binding of CREB in response to cAMP-induced phosphorylation is not a widespread mechanism of fasting-induced gene expression, although the accessibility of a small set of CREB sites may be regulated by secondary mechanisms such as chromatin remodeling.

Previous *in vitro* studies had suggested that CREB-DNA interactions were strengthened by CREB phosphorylation at S133, and that different classes of CRE sequences showed different degrees of phosphorylation-induced binding [7]. Our Western Blot analysis (Figure 1D) confirms that in a controlled feeding experiment, S133 phosphorylation of CREB is indeed specific to the fasted state, and therefore is likely to be involved in fasting-induced gene activation as previously hypothesized [2,6,44]. Although work by Koo and colleagues showed that S133 phosphorylation of CREB in liver was unchanged after an acute injection of insulin, used as a surrogate for the re-fed state [5], this manipulation does not mimic the physiological effects of re-feeding. Consistent with this notion, recent work has shown that insulin injection and re-feeding produce different results in terms of CREB co-factor recruitment and target gene activation [15]. Thus, our data support a model in which CREB is constitutively bound to its target genes, and fasting/cAMP signals induce changes in CREB phosphorylation and co-activator recruitment that promote gene expression from a subset of CREB-bound sites.

In the mammalian liver, the cAMP-induced phosphorylation of CREB S133 is known to enhance the recruitment of co-activators CREBBP, CRTC2, and EP300, while both CRTC2 and CREBBP are phosphorylated in response to insulin signaling to inhibit their interactions with CREB [5,16,45]. However, none of these co-activators have sequence specificity, as they are recruited to DNA indirectly by CREB and many other transcription factors. Furthermore, previous ChIP-chip studies have suggested that CREB phosphorylation on S133 occurs at the majority of CREB binding sites and is not specific for cAMP-inducible target genes [6]. Thus, other factors or genomic features must influence the cAMP-responsiveness of specific CREB target genes.

### Novel CREB target genes in mammalian liver

Previous genomic analyses of CREB DNA binding in liver cells were performed on human hepatocytes using promoter tiling arrays [6,14]. A systematic comparison of the target genes identified by our ChIP-seq study against the study by Zhang and colleagues [6], which included mapping of both gene symbols and homology mapping of gene regions, revealed a strikingly small overlap (only 14% of genes, Figure 3E). This undoubtedly reflects, in part, evolutionary differences between mice and humans. However, comparative ChIP-seq studies between mouse and human for other transcription factors have demonstrated much higher overlap of target genes (≥50%), even when the individual binding sites were not well conserved [38,46]. Thus, the lower than expected overlap observed here likely also reflects the lower sensitivity of the prior ChIP-chip study, which was limited to only proximal promoter regions known at the time. Our ChIP-seq study revealed sites in introns and distal upstream regions (Figure 3A), which were not included in the previous ChIP-chip array design. Furthermore, Zhang and colleagues estimated that their ChIP-chip study had only ~50% sensitivity among the proximal promoter regions [6].

Thus, our study reveals thousands of CREB target genes that are novel within the context of the mammalian liver. Roughly half of these genes were previously observed as CREB target genes in genome-wide studies of other cell types or predicted by bioinformatics searches for functional CRE motifs [6,32], but our study is the first to establish most of these genes as CREB targets in liver. The remaining genes, which total over 2,500, were not identified as CREB targets in any of the genome-wide studies surveyed here. Many of these genes, such as *Creld2*, *B4galt5*, and *Onecut1/Hnf6*, have no known connection to CREB in the literature, but have notably higher expression in re-fed livers compared to fasted livers. Overall, our analysis has revealed new functional roles for CREB, such as targeting ER stress genes induced by the opposing switch from fasting to feeding (Figure 5B, green bars).

### Functionally distinct subsets of CREB binding sites specify alternate responses to metabolic state

By integrating the CREB cistrome with fasting-dependent gene expression changes, we identified genomic features associated with the subset of CREB sites conferring cAMP-inducible activation to nearby genes. Our genome-wide analysis demonstrates that previously proposed elements, such as promoter TATA boxes [18], are only weakly associated with fasting-responsive CREB target genes. Our integrative analysis further revealed extensive co-localization at individual regulatory elements by both CREB and additional transcription factors CEBPB, NR3C1/GR, PPARA, and FOXA2.

Our results expand on targeted studies of individual gene promoters and suggest that synergistic interactions between transcription factors are likely to play a genome-wide role in the hepatic fed/fasted transcriptional switch. For example, Zhang and colleagues previously showed that liver-specific ablation of *Foxa2* attenuated *Pck1* induction in response to cAMP in primary hepatocytes [8]. CEBPB has been shown to directly activate *Pck1* expression in a manner dependent on the CREB binding site at the *Pck1* promoter [20]. Christoffels and colleagues have shown synergistic effects of NR3C1/GR agonist and cAMP on the activity of an enhancer containing both CREB and GR binding motifs upstream of *Cps1*[37]. Our results here suggest that such cross-talk occurs extensively within narrow regulatory regions throughout the genome, and is strongly associated with the specificity of the transcriptional response to feeding/fasting transitions.

Surprisingly, we found that CREB-binding is associated equally with feeding-induced genes as with fasting-induced genes. There is no known mechanism by which CREB might act as a transcriptional repressor. However, our results may reflect a feedback mechanism, in which some fasting-induced CREB targets are silenced prior to the 24 hr time point used in our experiment. Inducible cAMP early repressor (ICER), a repressive protein resulting from an alternate transcript of the CREB-related gene *Crem*, is a likely candidate for this mechanism [44], and our CREB cistrome confirms CREB binding at the alternate promoter of the ICER transcript (Additional file 1: Figure S3C). Alternatively, the subset of fasting-repressed genes might be responding to a combination of CREB and a Maf factor [43], as suggested by our *de novo* motif analysis (Figure 7G).

Another possibility is that genes with higher expression in re-fed livers are “feeding-induced”, rather than “fasting-repressed”. The increased expression of ER stress genes in re-fed livers supports this notion, as nutrient influx has been shown to increase ER stress signals in liver and other tissues [33,34]. Previous studies have shown that CREB competes for CRTC2 with the ER stress-induced Activating transcription factor 6 (ATF6), thereby providing antagonism between nutrient-influx and nutrient-deprivation signals [47,48]. Our novel observation that CREB also binds many ER stress-induced genes may indicate a more direct mechanism for this antagonism. Alternatively, CREB may play a role in the transduction of both signals, with genomic and epigenomic context determining the specificity of each response. The additional connection between CREB and ER stress revealed by our analysis is especially interesting given the clinical observation that chronic ER stress and overactive hepatic glucose production are both features of type 2 diabetes and obesity [10,49,50].

## Conclusions

Overall, our results demonstrate that CREB binding in the mammalian liver is constitutive with respect to metabolic state and widespread throughout the genome. The mechanisms contributing to fasting-inducible expression changes at CREB target genes required for glucose homeostasis are more complex than previously appreciated. The majority of CREB target genes are not induced by fasting and previously proposed mechanisms for specifying this response — e.g., site-specific dynamic occupancy [7] and interactions with TATA-containing promoters [18] — do not sufficiently explain fasting-responsive gene changes. The data presented above clearly show that CREB co-localization with the additional transcription factors CEBPB, NR3C1/GR, PPARA, and FOXA2 is specifically enriched around genes induced by fasting. Co-localization of these factors is significantly less frequent at CREB sites around genes with lower expression after fasting, suggesting a mechanistic difference between these two groups of regulatory elements. Our integrated CREB cistrome and fasting/re-feeding transcriptome provide essential tools for further investigating the role of transcriptional regulatory networks in the fasting response and other hepatic processes.

## Methods

### Animals

All mice used in this study were 8–12 week-old male C57BL/6 J mice. All experiments were conducted under a protocol approved by the IACUC of the University of Pennsylvania.

### Determination of blood glucose levels

Blood was sampled from the tail vein of fasted or re-fed mice, and glucose levels were measured with a OneTouch Ultra Glucometer (Lifescan Inc.).

### RNA isolation and quantitative RT-PCR analysis

Total RNA was extracted from livers collected from fasted and re-fed mice using the RNeasy Kit (Qiagen), then assayed for quantity and quality with the Agilent 2100 Bioanalyzer (Agilent Technologies). RNA was reverse transcribed using oligo (dT) and Superscript II reverse transcriptase (Invitrogen). The resulting cDNA samples were used as template for qPCR experiments performed with SYBR GreenER qPCR Supermix (Invitrogen) and the SYBR Green program on the Mx3000 Multiplex Quantitative PCR System (Stratagene). Reactions were performed in triplicate and normalized relative to the ROX reference dye. Median cycle threshold values were used for analyses. In each group, one sample had consistently higher threshold values for all reactions indicating poor RNA isolation quality, and was therefore excluded from analysis. Expression levels were normalized to those of Hypoxanthine guanine phosphoribosyl transferase (HPRT) as the internal control. Primer information is available at http://www.med.upenn.edu/kaestnerlab/reagents.shtml.

### Western blot analysis

Western blot analyses were performed as previously described [16] using anti-phospho-CREB (Cell Signaling, 87G3/#9198) and anti-CREB (Cell Signaling, 48H2/#9197) antibodies.

### Expression microarray analysis

200 ng of total RNA from individual each fasted and re-fed mouse livers (n = 4 per group) were amplified and labeled with Cy3 or Cy5 using Low Input Quick Amp Labeling Kit, two-color (Agilent, #5190-2306) with a dye swap experimental design. Labeled samples were purified using the RNeasy Kit (Qiagen) and hybridized overnight to the Agilent 4X44 Whole Mouse Genome Array. After hybridization the arrays were washed and scanned with the Agilent DNA Microarray Scanner G2565B. Median intensities of each array element were captured with Agilent Feature Extraction v10.5.1.1 and normalized by the print tip loess method in LIMMA [51]. Differentially expressed gene calls were performed by SAM [52] at 10% FDR and absolute fold-change cutoff of 1.5. Raw microarray data is available through GEO under accession number GSE45731.

### Chromatin Immunoprecipitation (ChIP)-sequencing procedure

Liver chromatin from fasted and re-fed mice was prepared as previously described [53]. Immunoprecipitations were performed and ChIP-Seq libraries were prepared as previously described [16], using anti-CREB (Santa Cruz Biotech, sc-186), anti-NR3C1/GR (mix of: Santa Cruz Biotech, sc-1004 and Thermo Scientific, PA1-511A), and anti-CEBPB (Santa Cruz Biotech, sc-150) antibodies. Libraries for each mouse liver were sequenced individually on an Illumina GAIIx. Raw sequencing data is available through GEO under accession number GSE45674.

### ChIP-seq peak calling

Reads for each individual ChIP-seq library were mapped to the UCSC mm8 reference genome using Illumina’s Eland pipeline. Redundant reads were discarded within each replicate. For each condition (fasted, re-fed), non-redundant reads from all five biological replicates were pooled into a single read set, and initial peak-calling was performed with HOMER v3.0 (5% FDR) [24], using a large pool of previous experimental inputs as the background control. To identify peaks that are not dependent on any single replicate, additional read subsets were created by pooling every possible combination of four replicates from each condition (fasted or re-fed), and peak-calling was repeated with the same parameters. Initial peak-calls from the 5-replicate pool were discarded if they failed to be called in any of the 4-replicate pools (SSI test). The entire SSI peak-calling procedure (using both the complete 5-replicate sets and the 4-replicate subsets) was also repeated with GLITR (5% FDR) [25] or MACS v1.4 (default parameters) with PeakSplitter v0.1 [26,54] in place of HOMER, for the purpose of comparing algorithm performance only. Venn diagrams of peak calls were generated with Cistrome [55]. HOMER peak calls passing SSI filter were divided into quartiles by average peak height across both conditions. Sites were randomly selected from each quartile for ChIP-qPCR validation. For each selected site, primers were designed and validated on input DNA to confirm high amplification efficiency. Sites for which efficient primers could be designed were subsequently tested for CREB enrichment in two biological replicates of chromatin from livers of 24 h fasted mice. Sites showing average CREB enrichment >2-fold compared to 18S rDNA control regions were considered as passing validation. Based on this ChIP-qPCR validation, we applied a final peak height threshold of 0.35 RPM to the set of remaining CREB sites.

### Differential binding analysis

To determine the extent of differential CREB binding between the fasted and re-fed conditions genome-wide, we first merged the fasted and re-fed SSI HOMER peak calls with average height > 0.35 RPM to a common set of 7,547 high-confidence binding regions. At each region, the peak height was computed individually for each replicate by extending reads to the average fragment length of 108 bp and computing the maximum value of the resulting stack height profile within each binding region. Peak heights were normalized to RPM for each replicate, and the normalized values were averaged to compute a single value for each condition. The raw number of tags from each replicate overlapping each high-confidence peak was tabulated and used for statistical analysis by EdgeR [30] with default parameters and 10% FDR after Benjamini-Hochberg p-value correction. For comparison to other transcription factor ChIP-seq data, the normalized peak height was computed at each of the 7,547 high-confidence CREB sites to assess co-localized binding, and peak height distributions were compared across functional subsets of CREB sites. ChIP-seq data for FOXA2 (GSE25836), PPARA (GSE35262), and CTCF (GSE36027) are previously published and available from GEO.

### Motif analysis

Initial identification of consensus CRE sequences in each binding region was performed by scanning the 100 bp sequence around each site for the octamer TGACGTCA with at most one mismatch, and the matching sequences were classified as “Full CRE” sites. The remaining regions were scanned for a perfect match to either pentamer half-site CRE (TGACG or CGTCA), and matching sequences were classified as “Half CRE” sites. The remaining regions without a match to either CRE sequence were classified as “Non-CRE” sites. *De novo* motif analysis was performed using HOMER v3.0 [24] on the 100 bp (for whole cistrome) or 500 bp (for regulation associated subsets) of sequence around each site. Background genomic regions were randomly selected using CisGenome [56] to maintain the general distribution of distances from known transcriptional start sites, and the HOMER parameter ‘-cpg’ was used to normalize foreground and background sites by CpG content. *De novo* motifs were filtered to significantly enriched motifs matching ≥ 10% of target sequences. Positional motif enrichment plots were computed using the HOMER histogram function with 10 bp resolution. We used the HOMER motif-scanning function with the known TATA motif from JASPAR [57,58] to classify all genes as TATA-containing or TATA-less. Specifically, we considered a gene to be TATA-containing if it contained a motif hit with score ≥ 6 within 250 bp of the annotated TSS position. This criteria showed clear enrichment of TATA motifs 20-40 bp upstream of TSS, and resulted in roughly 20% of genes called as TATA-containing, in agreement with previous assessments of TATA-containing promoters [18,59,60].

### Functional analysis

Binding regions were classified by their association with known gene architecture by comparing the exact center position of each region to the mm8 RefGene track downloaded from UCSC Genome Browser [61]. Region centers that were 2 kb upstream to 200 bp downstream of a RefGene TSS were classified as “Promoter” regions. Remaining region centers were classified based on overlapping RefGene introns, exonic coding regions, 5’UTRs (>200 bp from TSS), and 3’UTRs. All other regions were classified as “Intergenic”. CREB-bound genes are defined as any gene with a high-confidence CREB peak center within 10 kb upstream of the TSS or anywhere in the gene body. To compare to CREB target genes from previous genome-wide studies, we mapped gene lists onto our current RefGene annotations using both gene symbols and by overlapping genomic coordinates, to avoid any bias from updated gene annotations. For studies performed in human cells, we mapped the corresponding human promoter regions to the mouse genome using the UCSC LiftOver utility [61]. For comparison to DHS regions, we downloaded the DnaseI Digital Footprinting Hotspots track in 8-week old mouse liver from ENCODE [22,23] as mm9 coordinates, and mapped these regions to mm8 using LiftOver.

## Abbreviations

ATF6: Activating transcription factor 6; cAMP: Cyclic adenosine monophosphate; CEBPB: CCAAT/enhancer-binding protein beta; ChIP: Chromatin immunoprecipitation; ChIP-chip: ChIP coupled to genomic microarrays; ChIP-seq: ChIP coupled to high-throughput sequencing; CRE: cAMP response element; CREB: cAMP response element binding; CREBBP: CREB binding protein; CRTC2: CREB-regulated transcription co-activator 2; EP300: E1A binding protein p300; ER: Endoplasmic reticulum; FOXA2: Forkhead box protein A2; GR: Glucocorticoid receptor; HPRT: Hypoxanthine guanine phosphoribosyl transferase; ICER: Inducible cAMP early repressor; PPARA: Peroxisome proliferator activated receptor alpha; PKA: Protein kinase A; RPM: Reads Per Million; S133: Serine 133; SACO: Serial analysis of chromatin occupancy; SSI: Single sample independence; TSS: Transcription start site

## Competing interests

The authors declare no competing interests.

## Authors’ contributions

LJE, JL, and KHK conceived and planned the overall project. LJE performed all computational analyses. JL designed and carried out the CREB ChIP-seq experiment. LJE, SL, and DB designed and carried out CREB binding site validation and liver microarray experiments. DJS and MAL designed and carried out CEBPB and GR ChIP-seq experiments. All authors read and approved the final manuscript.

## Supplementary Material

Additional file 1 Supplementary tables and figures. Click here for file

Additional file 2 Summary tables of genes bound by CREB and regulated in fasting/re-feeding microarray experiment. Click here for file

Additional file 3 Summary table of all high-confidence CREB peaks, including peak height in each replicate, nearest TSS, and enrichment levels of additional transcription factors. Click here for file
