# Supplementary material for: Integrative genomic analysis of CREB defines a critical role for transcription factor networks in mediating the fed/fasted switch in liver
Source: BMC Genomics. 2013 May 17;14:337. doi: 10.1186/1471-2164-14-337 (PMC3671974; doi:10.1186/1471-2164-14-337)
Supplement: Additional file 1 — Supplementary tables and figures. [file 1471-2164-14-337-S1.pdf]

Supplementary Information for Everett LJ, Le Lay J, Lukovac S, Bernstein D, Steger DJ, Lazar MA, and Kaestner KH: Integrative genomic analysis of CREB defines a critical role for transcription factor networks in mediating the fed/fasted switch in liver. *BMC Genomics* 2013.

<u>**Table of Contents**</u>

Table S1	-	p2
Table S2	-	p3
Figure S1	-	p4
Figure S2	-	p6
Figure S3	-	p7
Figure S4	-	p8
Figure S5	-	p9
Figure S6	-	p10
Figure S7	-	p11

	Fasted	Re-fed
Replicate 1 Reads	9,837,202 (23%)	9,944343 (15%)
Replicate 2 Reads	9,539,204 (23%)	17,576,158 (26%)
Replicate 3 Reads	5,152,358 (12%)	10,792,771 (16%)
Replicate 4 Reads	7,593,038 (18%)	21,031,306 (31%)
Replicate 5 Reads	10,241,499 (24%)	9,013,609 (13%)
Total Reads	42,363,301	68,358,187
HOMER Peaks	39,308	14,792
Peaks Passing SSI	8,549 (22%)	6,123 (41%)
Peaks after qPCR Validation	6,835 (17%)	5,357 (36%)

Table S1: Overview of Read and Peak Counts. Per replicate and total read counts, along with number of peaks called by HOMER before and after filtering steps. Replicate read counts are shown with percentage of total reads for same condition, and filtered peak counts are shown with percentage of peaks called by HOMER for same condition.

Peak Coordinates (mm8)	Peak FC	Nearby Genes	Comments
chr1:95276058-95276168	3.37	Hdlbp	Adjacent to a peak with only 1.38x FC
chr11:78352445-78352553	0.45	Tnfaip1, Ift20	Adjacent to a peak with only 0.98x FC.
chr14:12914205-12914317	0.54	Psmd6	
chr14:64334718-64334826	0.45	Zfp395	
chr2:167551866-167551984	2.68	---	
chr4:49581043-49581153	4.33	---	
chr4:132247084-132247194	5.33	Fgr	
chr4:150448402-150448512	3.80	Camta1	Adjacent to a peak with 1.96x FC
chr7:16141534-16141644	4.94	---	
chr7:126486507-126486615	0.34	Slx1b, Bola2	
chr8:97068254-97068364	9.13	Mt1	Mt1 is significantly induced 5x in fasted livers
chr9:21821762-21821873	2.35	Zfp653, Gm16845	

Table S2: Differential CREB binding sites. The 12 CREB binding sites determined to be differentially enriched between fasted and re-fed livers by EdgeR (10% FDR). FC = fasted/re-fed fold change. Nearby genes are defined as all genes for which the CREB peak overlaps the gene body or 10kb upstream region. Adjacent peaks are defined as being within 500bp of the differential peak center.

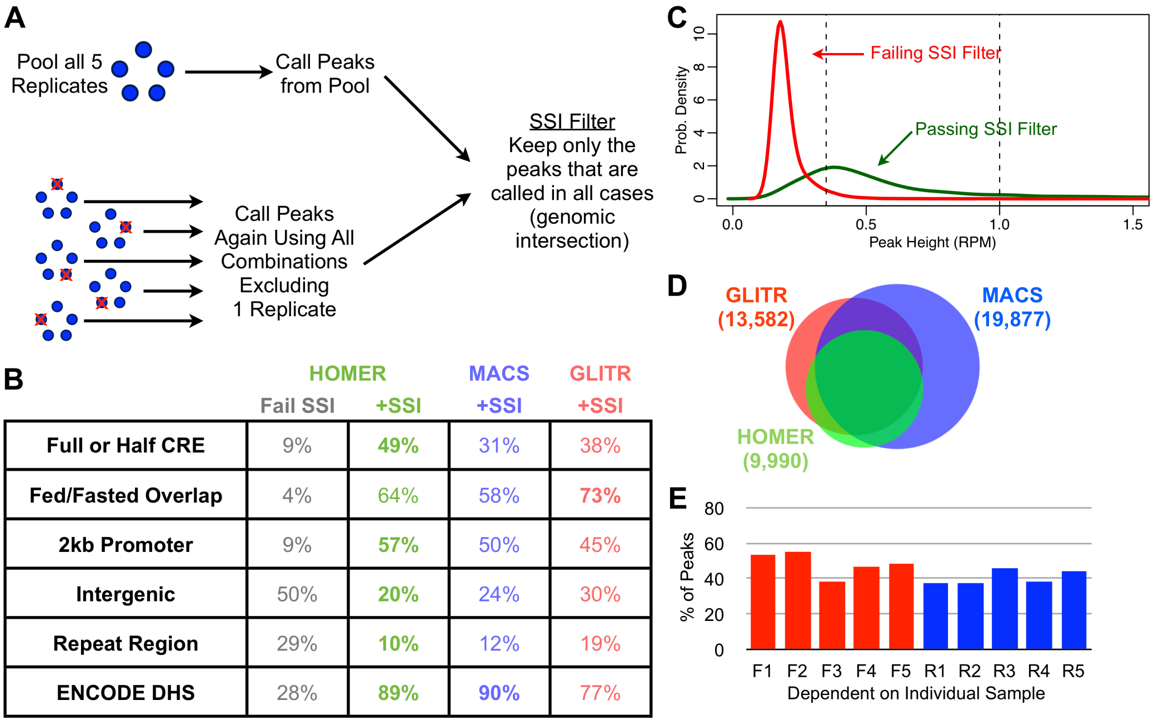

	HOMER Fail SSI	HOMER +SSI	MACS +SSI	GLITR +SSI
Full or Half CRE	9%	49%	31%	38%
Fed/Fasted Overlap	4%	64%	58%	73%
2kb Promoter	9%	57%	50%	45%
Intergenic	50%	20%	24%	30%
Repeat Region	29%	10%	12%	19%
ENCODE DHS	28%	89%	90%	77%

Figure S1: SSI Peak-Calling Strategy. A) Schematic of our strategy for filtering peaks based on reproducibility in five biological replicates sequenced separately. This strategy can be adapted to any number of replicates ≥ 3, and can be used with any peak-calling algorithm. **B)** Peaks containing CRE motifs or overlapping proximal promoters are consistent with known CREB biology and therefore serve as surrogate metrics inversely proportional to false positive rate. Repeat regions are more prone to sequencing artifacts and therefore can be used as a proxy proportional to false positives. Left-most column (gray) shows the rates of CRE motif occurrence, and alignment to proximal promoters, intergenic regions, and repeat regions, for 36,865 HOMER peak calls filtered by SSI strategy, demonstrating that the SSI filter removes sites more likely to be false positives based on surrogate metrics compared to 9,990 HOMER peak calls passing SSI filter (green column). Comparison between peak calls passing SSI from HOMER versus those from MACS and GLITR show that HOMER has the highest specificity estimated from surrogate metrics. **C)** Distribution of HOMER peak heights passing (green line) and failing (red line) the SSI filter. The dashed line at 0.35 RPM shows the empirically determined cutoff based on ChIP-qPCR validation. The dashed line at 1 RPM shows the arbitrary cutoff commonly used in ChIP-seq literature, which would result in the erroneous exclusion of many true CREB targets for our CREB ChIP-seq data. **D)** Venn Diagram of peak calls from different algorithms passing SSI filter, demonstrating that

HOMER calls the fewest algorithm-specific peaks. **E)** To assess the relative contribution of each replicate to the overall SSI procedure, we computed the percentage of peaks called from the initial pool of 5 replicates (red = fasted, blue = re-fed) that are lost when excluding each replicate.

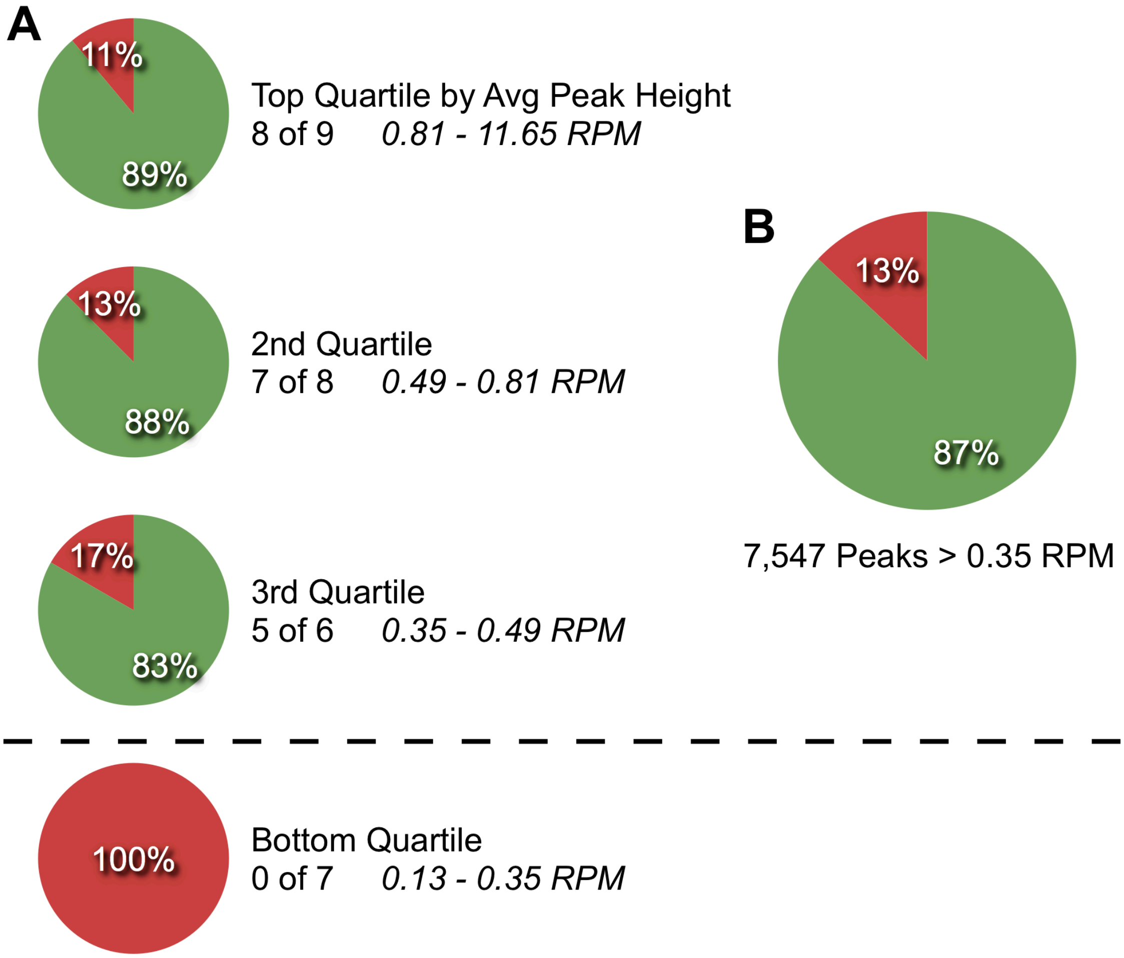

Figure S2: Peak Validation by ChIP-qPCR. Random peaks were selected from each quartile (by average peak height) of the HOMER peaks passing SSI. Peaks for which efficient primers could be designed were tested for CREB binding in fasted mouse liver in 2 biological replicates. Peaks with an average relative enrichment of ≥ 2-fold versus 18S were considered as passing ChIP-qPCR validation. **A)** Pass (green) and fail (red) rates for each quartile are shown as pie charts with the range of peak heights in that quartile. **B)** Pass/fail rates for all peaks above the 0.35 RPM cutoff (top 3 quartiles) are summarized in a single pie chart.

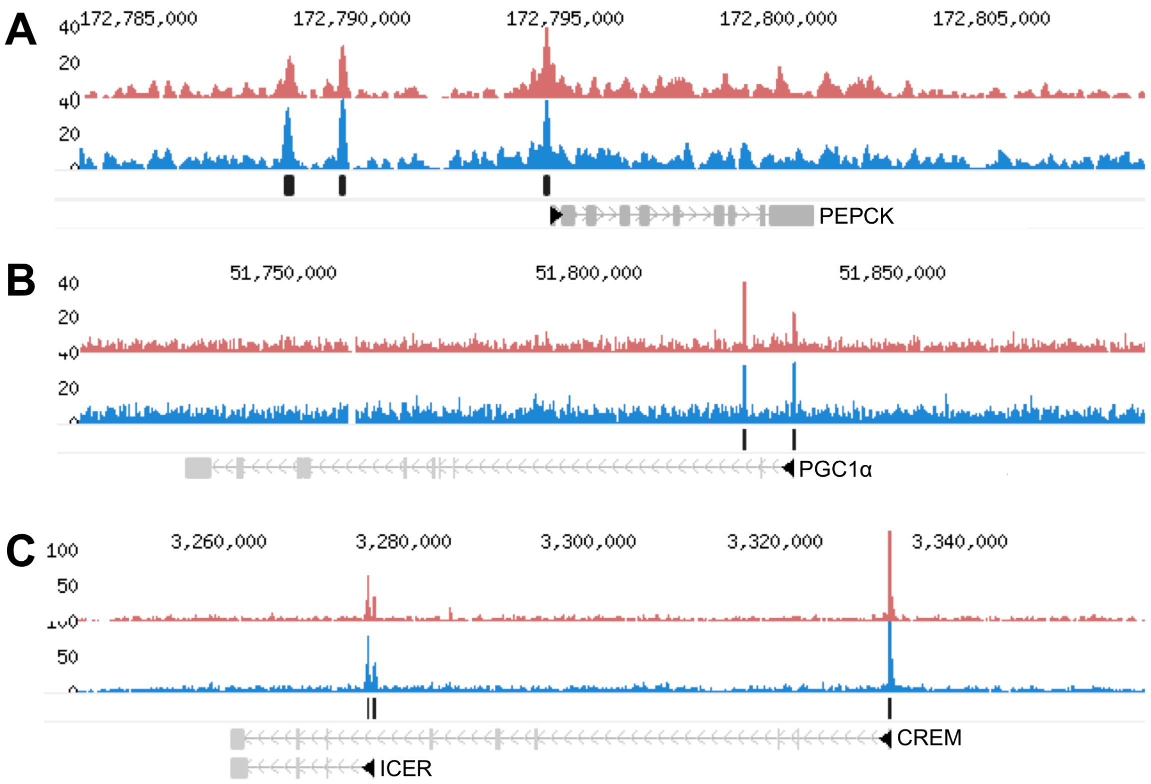

Figure S3: Confirmation of Known CREB Target Genes. Browser tracks showing CREB ChIP-seq profiles in fasted (red) and re-fed (blue) mouse liver, based on pooling of all replicates for each condition. Y-axis shows stack height in raw read count. Black marks below ChIP-seq profiles indicate high-confidence peak calls passing all filters. **A)** CREB binding at *Pck1* (PEPCK) promoter and distal upstream sites. **B)** CREB binding at *Ppargc1a* (PGC1α) promoter and intronic site. **C)** CREB binding to *Crem* promoter and internal promoter for truncated isoform ICER.

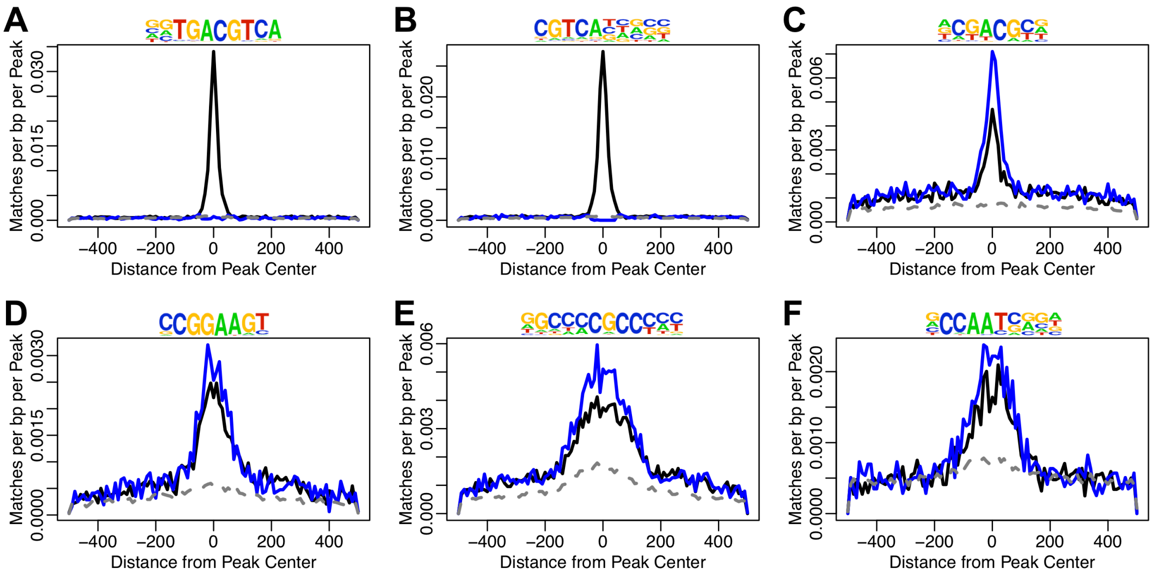

Figure S4: Positional motif analysis of CREB sites. CREB sites were separated by the sequence analysis shown in main manuscript Figure 4A (black lines = Full and Half CRE sites combined, blue lines = sites lacking canonical CRE, grey lines = randomly matched control sites). *De novo* motifs corresponding to those shown in main manuscript Figure 4C,D were scanned along the 1kb sequence surrounding each site and used to compute the match frequency in each 10bp segment. Positional plots are shown for: **A)** Full CRE motif, **B)** Half CRE motif, **C)** Degenerate CRE motif, **D)** ETS family motif, **E)** GC-Box/Sp1 motif, and **F)** NFY/CCAAT motif.

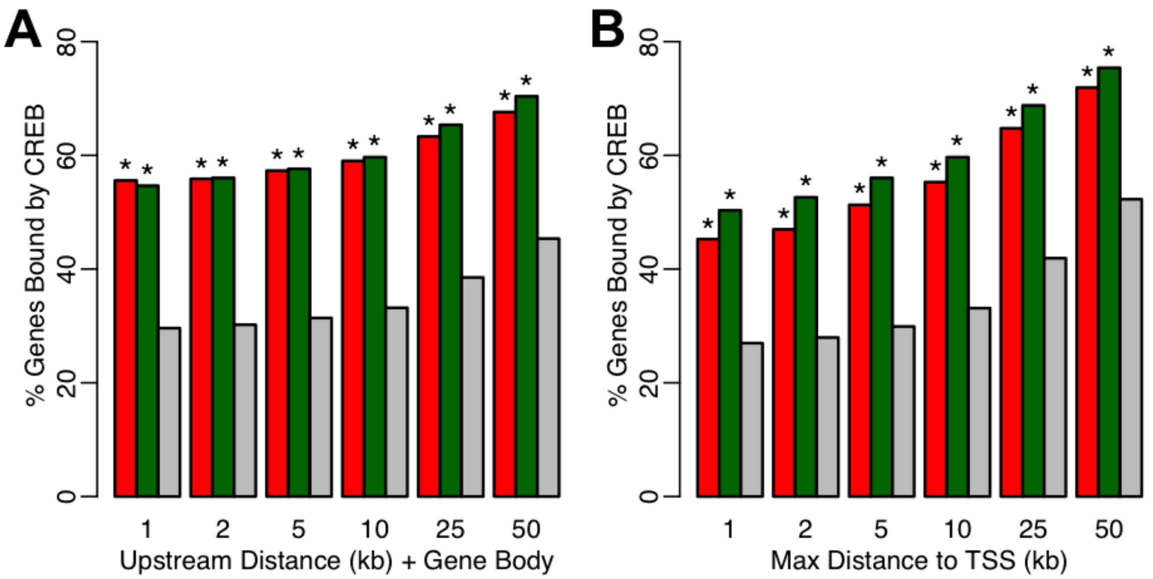

Figure S5: Enrichment of CREB binding around fasting-responsive genes at varying distance thresholds. Bar graphs showing the percentage of genes in each category (fasting-induced in red, fasting-repressed in green, and all genes on array in gray) that have a CREB binding site within the specified distance, either **A)** upstream of the TSS, or anywhere in the gene body, or **B)** within the specified distance of the TSS in either direction. The enrichment of CREB is significant at all thresholds shown, using either association model, although in both cases the background rate rises substantially using distances above 10kb. *p-value < 1E-16 by Fisher's Exact Test versus the corresponding threshold in background.

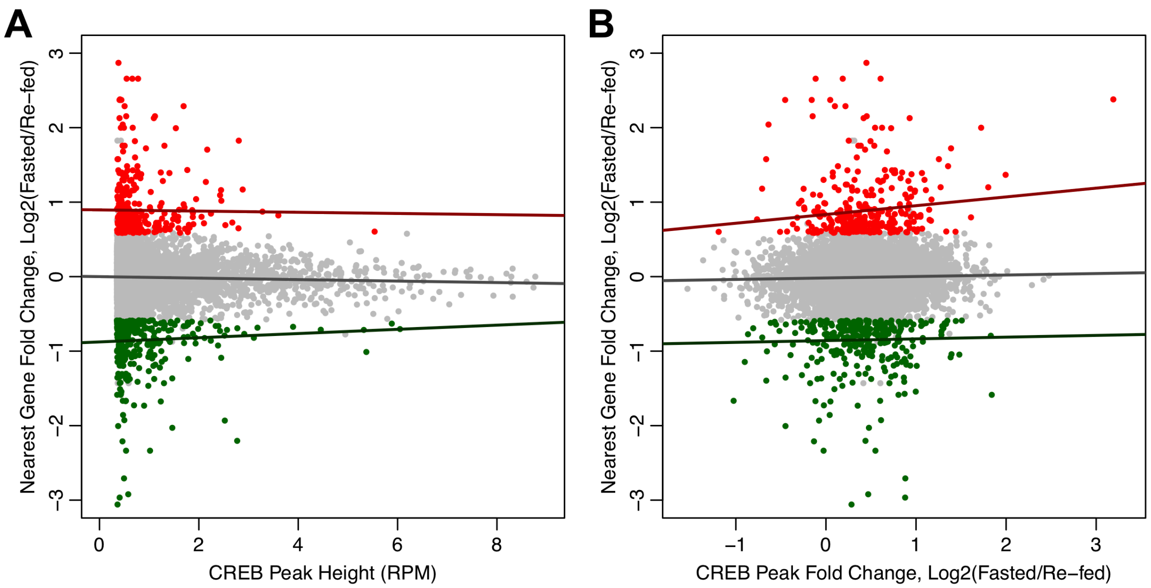

Figure S6: CREB binding strength does not correlate with feeding/fasting-inducibility of target genes. CREB peaks within gene bodies or 10kb upstream regions were assigned to the nearest TSS. Peaks assigned to significantly up-regulated and down-regulated genes are colored in red and green, respectively. Peaks assigned to non-regulated genes are colored in grey. **A)** Average peak height from both the fasted and re-fed livers was plotted against the log2(fasted/re-fed) ratio of the assigned gene expression. **B)** Log2(fasted/re-fed) ratio of CREB peak height was plotted against the log2(fasted/re-fed) ratio of the assigned gene expression. Best fit lines are shown for each colored group of points, and all groups in both plots had Pearson Correlation Coefficients weaker than +/- 0.1.

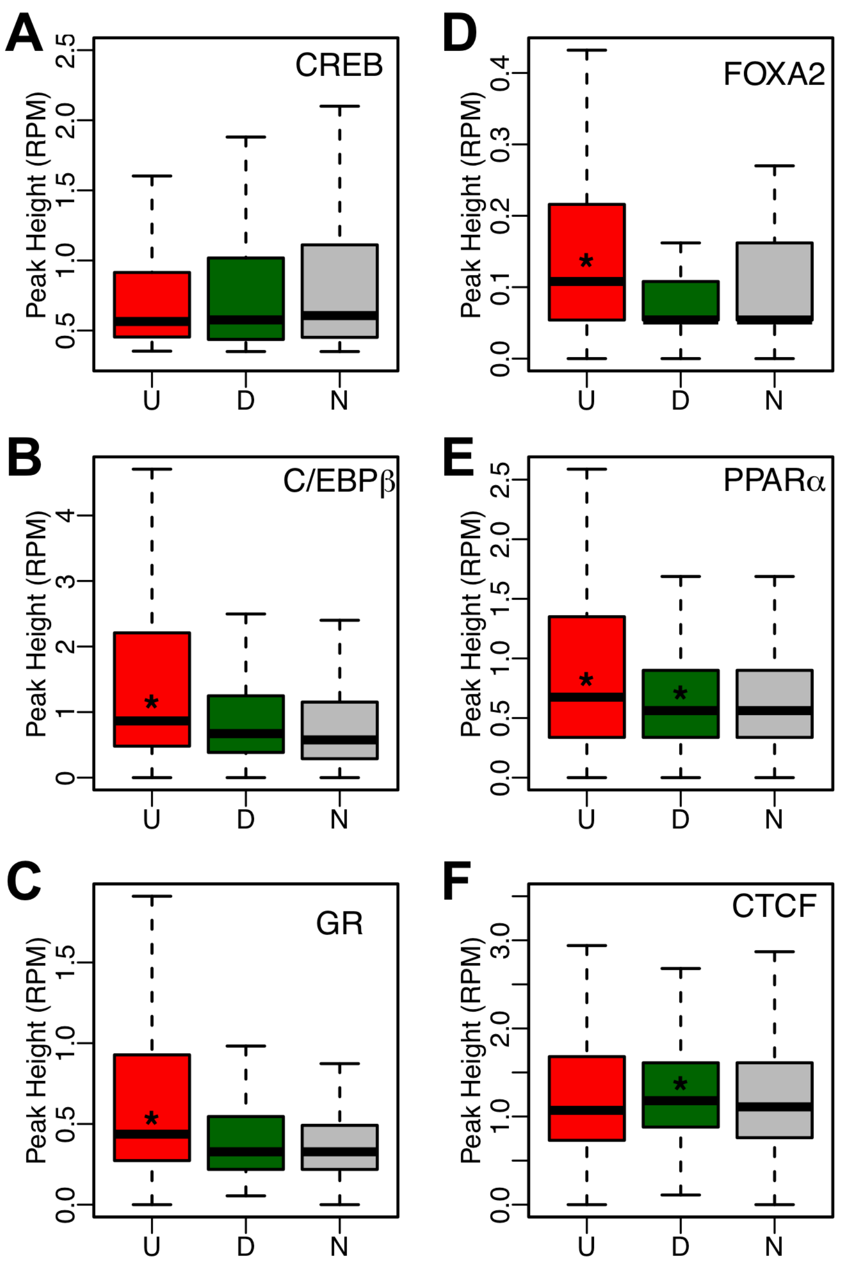

Figure S7: Distributions of peak heights for additional transcription factors at CREB sites. CREB sites were separated into groups as described in main manuscript Figure 7: 'U' = sites associated with up-regulated genes (red), 'D' = sites associated with down-regulated genes (green), 'N' = sites associated with non-regulated genes (gray). Boxplots show distributions of peak heights at each group of CREB binding sites for **A)** CREB, **B)** C/EBPβ, **C)** GR, **D)** FOXA2, **E)** PPARα, and **F)** CTCF. *p-value < 0.05, Mann-Whitney test compared to 'N' group.
